# Implementation of the nutrition care process on mortality risk in critically ill COVID-19 patients – a registry longitudinal cohort study

**DOI:** 10.1080/07853890.2025.2598901

**Published:** 2025-12-08

**Authors:** Lindsay Woodcock, Alexa Steiber, Zhongqui Fan, Lauren E. Louck, Alison Steiber, Constantina Papoutsakis, Taylor C. Wallace

**Affiliations:** ^a^ Research, International and Scientific Affairs, Academy of Nutrition and Dietetics, Chicago, IL, USA; ^b^ School of Public Health, Division of Epidemiology and Community Health, University of Minnesota, Minneapolis, MN, USA; ^c^ Gerald J. and Dorothy R. Friedman School of Nutrition Science and Policy, Tufts University, Boston, MA, USA; ^d^ Think Healthy Group, LLC, Washington, DC, USA; ^e^ Department of Nutrition and Food Studies, George Mason University, Washington, DC, USA; ^f^ School of Medicine and Health Sciences, George Washington University, Washington, DC, USA

**Keywords:** COVID-19, nutrition care process, death, intensive care units, nutritionists, dietetics

## Abstract

**Background:**

COVID-19 impacts the nutritional status of patients. There is limited longitudinal research on the impact of nutrition care provided to COVID-19 patients in the intensive care unit (ICU).

**Objective:**

We assessed the real-world effectiveness of the nutrition care process (NCP) used by registered dietitian nutritionists (RDNs) on mortality in COVID-19 ICU patients.

**Methods:**

RDNs in 8 ICUs throughout the United States utilized the Academy of Nutrition and Dietetics Health Informatics Infrastructure (ANDHII) registry to document standard care practices, per the NCP, used with COVID-19 patients admitted from April 2021 to April 2022. Mortality, the primary outcome, was reported at the last visit. A multivariable logistic regression model, adjusted for sex, age, body mass index (BMI), and number of visits, estimated mortality risk as a function of the NCP problem status (improving/resolved).

**Results:**

Participants (*N* = 90) were mostly male (68.2%), with a mean age of 57.2 ± 15.7 years. Participants’ baseline c-reactive protein (CRP) levels were high (55.9 ± 74.2 mg/L) and about two-thirds were obese (63.7%), per BMI. 33 patients died over the study duration. The most common NCP problems identified were related to inadequate energy and protein intake, with 62.5% resolved through enteral nutrition interventions. Improvement and resolution of the NCP problem by the RDN was associated with 86% (OR: 0.14, 95% CI: 0.03–0.52, *p* = 0.004) and 89% (OR: 0.11, 95% CI: 0.01–0.67, *p* = 0.023) reduced risk of death, respectively.

**Conclusion:**

We show for the first time in a real-word setting that implementing the NCP to identify and address nutritional problems during ICU admission is associated with lower mortality risk in critical COVID-19 patients. Due to limitations in longitudinal registries and use of logistic models, future larger-scale cohorts and clinical trials are needed.

## KEY MESSAGES

- Improvement or resolution of the documented NCP problem was associated with a 86% and 89% reduced risk of death in COVID-19 patients admitted to the ICU, respectively.
- The most frequently identified NCP problems were related to inadequate energy and protein intake, with 62.5% of these improving or resolving as a result of enteral nutrition interventions implemented by RDNs.

## Introduction

COVID-19, caused by severe acute respiratory syndrome coronavirus 2 (SARS-CoV-2), is a highly contagious infectious disease that can rapidly escalate to respiratory failure and death [1,2]. Age, male gender, obesity, diabetes, hypertension, immunosuppression, and organ failure have all been identified as risk factors related to disease severity [3–5]. Augmented concentrations of C-reactive protein (CRP) and proinflammatory cytokines such as interleukin-6 have also been widely reported to be associated with increased severity and mortality in retrospective and cross-sectional cohort studies [6,7]. An individual’s nutritional status and diet can modulate inflammation and immune function and therefore may have the potential to impact outcomes of COVID-19 in the intensive care unit (ICU) setting [8]. Even though younger individuals have a decreased likelihood of critical care admission, obesity alone has been associated with a two fold increased risk [9]. Immune cells in general show high energy expenditure and the nutritional demand is dramatically increased during infection and immune system activation [10–12]. Growing evidence suggests that increased intake of certain nutrients above current recommended levels may help optimize immune functions including improving defense function [13]. COVID-19, like other infectious diseases that trigger a strong immune response, has an increased nutritional demand on the body due to acceleration of cell metabolism [14]. However, life-threatening hypoxemia often requires a delay of enteral nutrition [15].

Several researchers and scientific groups, notably the Society for Critical Care Medicine (SCCM), and the American Society for Parenteral and Enteral Nutrition (ASPEN) have re-emphasized generalized recommendations for nutrition therapy in critically ill adult patients with COVID-19 [16–18]. The European Society for Clinical Nutrition and Metabolism (ESPEN) has developed updated guidelines that are specific to the needs of patients who are critically ill with COVID-19 [19]. It is common for patients in the ICU and post-ICU hospitalization to not receive their full nutritional requirements [20–22]. Multiple cohort analyses have shown initiation of nutritional support to be slow and very often never reaches recommended targets for total calories, macronutrients, and micronutrients during the ICU stay [20]. It has been estimated that >40% of ICU patients are not fed during the first day [20]. Resulting malnutrition has been long linked to adverse health outcomes in the ICU setting [23]. A retrospective cohort study of adult COVID-19 inpatients (*N* = 4,311) showed those with malnutrition had a 76% (OR: 1.76, 95% CI: 1.34–2.30, *p* < 0.0001) higher odds of mortality and a 105% (β: 0.72, 95% CI: 0.587– 0.854, *p* < 0.0001) longer hospital length of stay [24]. Nutritional status, ascertained predominantly from retrospective review of electronic medical record (EMR) data, appears a relevant factor influencing outcomes in patients with COVID-19; these data indicate that a patient’s nutritional status and preventing or treating malnutrition are cornerstones to reducing mortality and complications in the critical care setting. While total calorie and protein intakes are the most common and widely associated nutritional deficits contributing to increased mortality, length of stay, and other complications, other macronutrients such as omega-3 fats and micronutrients including vitamins A, C, D, E, iron, magnesium, zinc, and copper have well established immunomodulatory effects [25,26], however, there is a dearth of longitudinal information on how early nutrition support, standard care practices, and the widely adopted Nutrition Care Process (NCP) influences progression and mortality outcomes in COVID-19 patients admitted to the ICU. Despite the high prevalence of malnutrition in critically ill patients, particularly those with COVID-19, clinical outcomes are likely affected by quick and nonuniform assessment; calorie intake, protein intake, and weight change are amongst the only key nutrition-related metrics routinely monitored longitudinally in ICU patients with COVID-19 [27,28]. This likely reflects the global shortage of trained RDNs in the ICU setting, absence of meaningful clinical nutrition training in medical curricula, and lack of awareness of international clinical practice guidelines for nutrition support [29–32]. The NCP is a systematic approach to providing nutrition care. It consists of four distinct, interrelated steps: nutrition assessment, nutrition diagnosis, nutrition intervention, and nutrition monitoring/evaluation [33,34]. We previously described nutrition care patterns in COVID-19 ICU patients and demonstrated that RDNs have the potential to resolve nutrition problems in this population [35]. These cross-sectional data [35] informed the design and implementation of the present Nut**r**itional Car**e** Practice**s** and S**t**atus **o**f SA**R**S-CoV-2 Pati**e**nts (RESTORE) registry longitudinal cohort, which aims to assess the real-world effectiveness of implementing the NCP on mortality in critically ill adult patients with COVID-19.

## Materials and methods

### Study design

Methods and findings from The RESTORE Study are reported according to the Strength of Reporting Observational Studies in Epidemiology (STROBE) guidelines [36]. The RESTORE Study is an observational longitudinal cohort study that utilized the Academy of Nutrition and Dietetics Health Informatics Infrastructure (ANDHII), a web-based registry platform [35,37,38], to document de-identified data from COVID-19 patients admitted to 8 ICUs throughout the United States between April 2021 and April 2022. RDNs providing care to patients in critical care hospital units across the United States were recruited in a rolling fashion starting in February 2021 through advertisement in the Academy of Nutrition and Dietetics (Academy) and affiliated group listservs and social media posts. Effort was made to include hospitals of different sizes and from different regions and community settings of the United States. Over sixty-five hospital sites responded to recruitment advertisements and 8 of these participated in the study. This was a multicenter study which used the Institutional Review Board (IRB) Office of George Mason University as the IRB of Record. This IRB determined the study does not meet the definition of human subject research activities (IRB # 1607972-1). All research activities adhered to the Declaration of Helsinki. Local site IRBs either accepted this determination or completed their own independent IRB review before RDNs were allowed to enter data on their ongoing standard care practices, per the NCP. Participating RDNs also completed self-paced online trainings related to (1) appropriate NCP documentation, (2) use of the ANDHII platform, and (3) Research Ethics. RDNs participating in the study provided written informed consent. De-identified data from adult patients (18+ years of age) who had been diagnosed with COVID-19, admitted to the ICU, and received nutrition care from a participating RDN was entered into the ANDHII platform.

### Nutrition care data entry

RDNs used the NCP Terminology (NCPT), a standardized language, to describe the care provided to patients [35,39]. Due to the ‘real-world’ nature of registry study design and the IRB determination of non-human subjects research, each RDN was free to document their usual patient care [38]; investigators were thus precluded from mandating that specific variables or practices be documented throughout the study. Participating RDNs were provided a list of suggested NCPT, which included indicators commonly recorded in the documentation of this patient population at the time the study initiated in April 2021. This list was reached by consensus with input from nutrition researchers, RDNs, and other clinicians knowledgeable of the ICU setting. Available in the NCPT are terms to record characteristics of patients, including demographics, anthropometric data, laboratory values, and nutrition-related indicators, including assessments of energy and protein intake. If the patient was seen for a follow-up visit by the RDN, this was entered into ANDHII in separate follow-up notes. For each patient and in each visit, RDNs recorded three components of a nutrition diagnosis: the nutrition problem, etiology, and signs and symptoms. RDNs recorded the status of each nutrition problem at each visit (active-unresolved, active-improving, and resolved) and each nutrition diagnosis for the patient was followed as an episode of care (collection of continuous nutrition visits addressing the recorded individual patient’s nutrition diagnoses). Data were categorized for each visit starting with visit 1 (i.e. the baseline consult with the RDN), visit 2, visit 3, and so on; however, the time difference between visits is not known due to the HIPPA compliance of the ANDHII platform. RDNs were able to record multiple nutrition diagnoses during their course of care, thus this convenience sample (largely) does not reflect repeated measures data. RDNs were asked to document whether or not the patient was deceased at the patient’s last visit; mortality served as the primary outcome of the study. Patients were tracked for ventilation status as being ventilated during the course of RDN care documented in ANDHII, not ventilated, or unknown. Two patients whose ventilation was documented as removed at the first or second visit were considered not ventilated during the course of care. Participating RDNs were offered a $25 gift card for entering at least 10 patients into ANDHII.

### Data management

Data was extracted from ANDHII, then classified according to NCP chain components, organized, and cleaned using Microsoft Excel. Specifically, nutrition problems documented for each patient were coded (i.e. assigned a unique code to track the problem across visits for the patient). The status of the problem (new, active – improving, active – unresolved, resolved, discontinued) was recorded by the RDN when entering patient data. Each instance of a new nutrition problem was counted as a nutrition problem encounter. The status of the nutrition problem was followed across visits. If a nutrition problem was documented as ‘new’ multiple times within the patient’s care, it was considered as a new nutrition problem.

## Statistical analysis

Continuous variables are described as mean ± SD, and dichotomous variables as the number (n) and percentage (%) of the population. To assess the association between status of the nutrition problem and risk of mortality, we constructed a multivariable logistic regression model, adjusting for the following *a priori* covariates: sex, age, BMI, and number of RDN patient visits. A quasi-complete separation was checked for all covariates. All statistical analyses were performed using R software (version 4.2.1) and RStudio 2022.12.0 + 353 (RStudio, PBC). Statistical significance was set as *p* ≤ 0.05.

## Results

In total, 90 patients diagnosed with COVID-19 in the ICU were included in the study (Table 1). The majority of patients were middle-aged or older (57.2 ± 15.7 y), mostly male (∼68%), and obese (BMI >30 kg/m^2^) (∼64%). C-reactive protein (CRP) levels were high (55.9 ± 74.2 mg/L) and total energy intake within the last 24-hours low (607 ± 586 kcal) among participants upon entry to the ICU. Approximately 58% of patients required mechanical ventilation at some timepoint and 33 died during the study.

**Table 1.** Participant baseline characteristics at the first visit (*N* = 90).

Characteristic	
Sex	
Female	27 (31.8%)
Male	58 (68.2%)
Age (years)	
Mean (SD)	57.2 (15.7)
BMI, n (%)	
<18.5 – Underweight	2 (2.3%)
18.5–24.9 – Normal weight	10 (11.4%)
25.0–29.9 – Pre-obesity	20 (22.7%)
30.0–34.9 – Obesity class I	21 (23.9%)
35.0–39.9 – Obesity class II	21 (23.9%)
≥40 – Obesity class III	14 (15.9%)
Biomarkers, mean (SD)	
C-reactive protein (mg/L)	55.9 (74.2)
Glucose (mg/dL)	184 (59.6)
Sodium (mEq/L)	138 (6.53)
Potassium (mEq/L)	4.37 (0.75)
Calcium (mg/dL)	8.11 (0.68)
Magnesium (mmol/L)	1.93 (1.79)
Albumin (g/dL)	2.98 (0.338)
Total energy intake in 24 h (kcal)	607 (586)
Ventilation status, n (%)	
Ventilated	51 (58.0%)
Morality status, n (%)	
Death	33 (36.7%)

**Figure 1.**
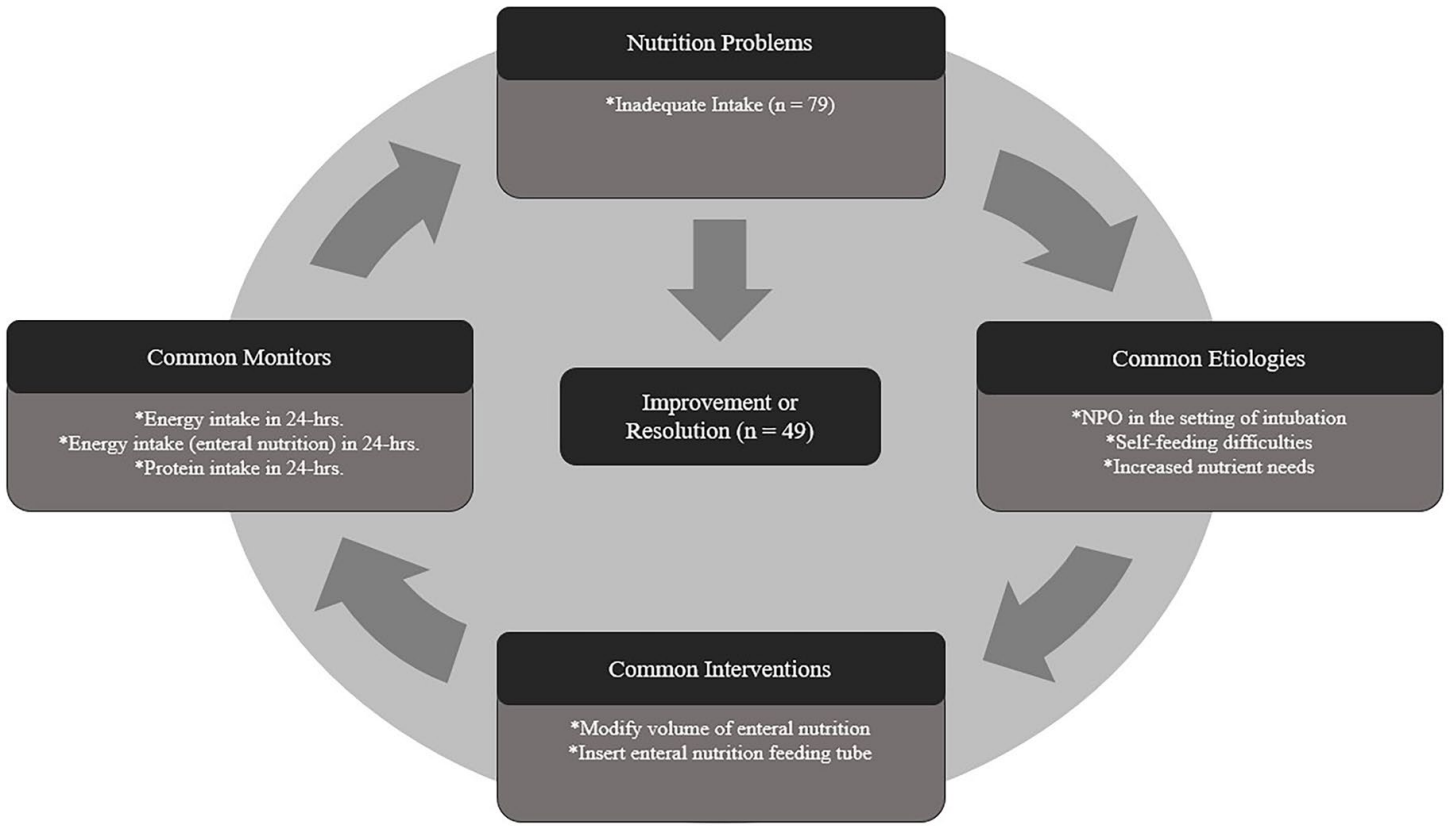
The most common etiologies, interventions and monitors recorded when ‘*inadequate intake*’ was documented as the nutrition problem at baseline (*n* = 79). ICU = intensive care unit; NPO, nil per orum

An initial visit with at least one follow-up visit were recorded by RDNs for the majority of patients (93%). A total of 7,010 NCP terms were used by RDNs. A total of 133 nutrition problems, including 93 related to inadequate intake (energy, protein-energy, oral, and protein), were documented across a total of 322 RDN patient visits. The majority (65%) of nutrition problems related to inadequate intake were later documented as being resolved or improved over the course of the study. Inadequate energy, protein-energy, oral and protein intakes were either resolved or improved in 64%, 42%, 94% and 88% of documented cases over time. A 53% favorable increase was noted between the initial and final total energy intake in 24 h (from 864 ± 716 to 1,305 ± 739 kcal, *p* = 0.0062). Figure 1 displays the most common etiologies, interventions, and monitors documented when inadequate intake was recorded as the baseline nutrition problem. Of note, the cohort did not experience weight loss from the first to final RDN visit during the course of nutrition care (99.1 ± 33.2 vs. 98.3 ± 29.2 kg, *p* = 0.871).

A total of 73 patients were included in the multivariable adjusted logistic regression model that assessed the impact of the NCP on mortality. Patients whose nutrition problem was documented as ‘active-improving’ showed an 86% (OR: 0.14, 95% CI: 0.03–0.52, *p* = 0.004) reduced risk of mortality, compared to those documented as ‘active-unresolved’. Likewise, patients whose nutrition problem was documented as ‘resolved’ showed an 89% (OR: 0.11, 95% CI: 0.01–0.67, *p* = 0.023) reduced risk of mortality, compared to those documented as ‘active-unresolved’ (Table 2).

**Table 2.** Status of the NCP problem and risk of mortality in patients.

	OR (95% CI)	p-value
NCP problem^a^		
Active – Unresolved	–	–
Active – Improving	0.14 (0.03–0.52)	0.004
Resolved	0.11 (0.01–0.67)	0.023
Age	1.04 (1.00–1.09)	0.075
Sex		
Male	–	–
Female	1.94 (0.61–6.35)	0.3
BMI weight classification		
Normal weight – (18.5–24.9)	0.07 (0.00–1.01)	0.071
Overweight (25.0–29.9)	0.98 (0.12–8.14)	>0.9
Obesity – Class I (30.0–34.9)	1.09 (0.16–7.62)	>0.9
Obesity – Class II (35.0–39.9)	0.52 (0.08–3.21)	0.5
Obesity – Class III	–	–
# Patient visits by RDN	1.11 (0.83–1.53)	0.5

^a^ Adjusted for sex, age, BMI, and number of patient visits by the RDN.

NCP = Nutrition Care Process, OR = odds ratio, RDN = Registered Dietitian Nutritionist.

## Discussion

Nutrition care is essential in the management of COVID-19 patients in the ICU and to our knowledge, this the first longitudinal registry study to report on the impact of NCP delivered by RDNs on patient mortality. To those in the field, our results may not be surprising, as COVID-19 outcomes and severity have been associated with suboptimal nutrition-related indicators (e.g. obesity) since the start of the global pandemic in 2020. As previously stated, infectious disease and immune system activation exerts an increased nutritional demand on the body [14]. Malnutrition is a common issue in the ICU setting. A recent systematic review showed malnutrition to be present in 38% to 78% of ICU patients and be associated with increased risks of morbidity, mortality, and hospital-related cost [40]. Various protocols and guidelines have been published by specialized scientific societies that emphasize the importance of ensuring adequate protein and energy intake in COVID-19 patients by using nutrition support if nutrition needs cannot be met orally [15,17,18]. Economic impact models show in-hospital nutrition support to be a cost-effective way to reduce readmissions, lower the frequency of hospital associated infections, and improve survival rate [41]. Our study reinforces the universal need for RDNs and standardized NCP in the critical care setting.

Other longitudinal studies from across the globe reinforce our findings. A similar multicenter 9 site registry study in Australia found the majority of COVID-19 patients being seen by a dietitian in the ICU were provided enteral nutrition (65%) and ventilated patients were more likely to receive dietetic care [42]. A prospective observational study in the United Kingdom showed dietetic-led interventions prevented further weight loss in COVID-19 patients in the ICU; dietetic-led interventions were required for all patients (*N* = 452) since the ICU nutrition protocol did not meet their nutritional needs [43]. Of note, weight loss did not occur during the RESTORE study, which likely reflects early active involvement of RDNs. In Ethiopia, a prospective cohort study of COVID-19 patients admitted to selected COVID-19 Isolation and Treatment Centers showed rate of survival to be to be better among well-nourished patients [44]. The COVID-19 pandemic resurfaced the longstanding need for enhanced nutrition support in ICU patients through systematic approaches like the NCP.

Our study has several limitations including a smaller sample size. Due to the nature of registry design, participants in our study represent a convenience sample that is not nationally representative; sampling bias may be present. The small sample size and differences in NCP documentation by enrolled RDNs impeded us from estimating effects of specific nutrition problems; this also limited us to the smaller number of covariates adjusted for in the model. The use of logistic regression, while most appropriately used for this type of non-repeated measures data, likely over-inflates the actual impact of the NCP on mortality. On the other hand, our study had several strengths, including the assessment of real-world data that reflects RDN practices within the ICU. The RDNs represented a diverse range of ICU settings across the United States; in particular, RDNs consistently documented and monitored energy and protein intake of patients as crucial to their survival, which gives us confidence that our findings are likely to hold true in a larger sample. Follow-up data was recorded on almost all patients (93%), which allowed for the investigation of nutrition problem resolution and improvement.

This research demonstrates the need for larger registry studies and clinical trials that evaluate the impact of the NCP on outcomes in COVID-19 and non-COVID-19 patients, since nutrition problems such as inadequate energy and protein intakes are common among patients in critical care.

## Conclusions

We show for the first time in a real-word setting that implementation of the NCP to identify and address nutritional problems early during ICU admission is associated with a lower risk of mortality in critically ill patients with COVID-19; however, due to inherent limitations in longitudinal registry study design and use of logistic models, future larger-scale cohorts and clinical trials are needed to fully confirm these findings.

## Data Availability

Raw data were generated by the Academy of Nutrition and Dietetics. Derived data supporting the findings of this study are available from the corresponding author upon reasonable request.
